# Natural Tyrosinase Inhibitors from *Lycopodium japonicum*

**DOI:** 10.3390/molecules30194024

**Published:** 2025-10-09

**Authors:** Zeng-Yue Ge, Ya-Qing Wang, Qi-Bin Yang, Xian-Yun Yan, Lei Wu, Min Zhang, Lin-Fu Liang

**Affiliations:** 1 School of Chemistry and Chemical Engineering, Central South University of Forestry and Technology, 498 South Shaoshan Road, Changsha 410004, China; 2 School of Forestry, Central South University of Forestry and Technology, 498 South Shaoshan Road, Changsha 410004, China

**Keywords:** *Lycopodium japonicum*, phytochemicals, serratane triterpenes, tyrosinase inhibitory activity, molecular docking

## Abstract

Natural tyrosinase inhibitors are an important group of compounds with cosmetical and medicinal applications. With the aim of finding new types of natural tyrosinase inhibitors from ornamental and medicinal plants, *Lycopodium japonicum* was selected and studied. As a result, fifteen structurally diverse secondary metabolites **1**–**15** were isolated and identified. Their chemical structures were identified by analysis of their spectral data and compared with those reported in the literature. In the tyrosinase inhibitory bioassay, five phytochemicals, **4**, **12**, **13**, **14,** and **15,** exhibited significant inhibitory effects, with half-maximal inhibitory concentration (IC_50_) values ranging from 1.46 to 6.82 mM. Additionally, molecular docking studies disclosed that Lys376, Lys379, Gln307, and other amino acid residues played key roles in the potential binding interactions between the active compounds and the tyrosinase. These findings suggest that the species *L. japonicum* is a warehouse of natural tyrosinase inhibitors.

## 1. Introduction

Tyrosinase is a multifunctional enzyme, which is responsible for the oxidation of phenolic compounds [1]. For humans, this enzyme plays a key physiological role because its dysregulated expression can lead to various skin disorders, such as melanoma, freckles, chloasma, and vitiligo [2,3]. Due to its crucial role in melanogenesis, tyrosinase inhibitors are regarded as an important group of compounds with promising cosmetic and medicinal values, such as skin tone enhancement and depigmentation [1,4].

*Lycopodium japonicum* belongs to the family Lycopodiaceae [5], which is distributed across Japan, China, India, and also Southeast Asia [6]. For a long time, this plant has been used as a medicinal material for the treatment of arthritic pain, quadriplegia, dysmenorrhea, contusions, and other health problems [6]. Nowadays, it is also used as an ornamental plant, due to its evergreen and beautiful branches and leaves. Previous phytochemical investigations of this plant disclosed that *Lycopodium* alkaloids [7,8,9,10] and serratane triterpenes [11,12,13,14] are two major groups of chemical constituents from *L. japonicum*. In addition, lignans [15], sterols [16], and other compounds are minor groups in the chemical composition of *L. japonicum*. These compounds exhibit an array of biological activities, including neuroprotective [7], anti-renal fibrosis [8], acetylcholinesterase inhibitory [17,18], cytotoxic [12], anti-inflammatory [19,20], anti-HIV-1 [18], and α-glucosidase inhibitory [17,21] activities. However, the tyrosinase inhibitory effect of the plant has not been investigated yet.

In our continuing efforts to search for bioactive substances derived from ornamental and medicinal plants for cosmetic products [22,23], the plant *L. japonicum* was studied. This work is intended to explore the non-alkaloidal constituents of the titular species with tyrosinase inhibitory activity in vitro and in silico.

## 2. Results

### 2.1. Structure Elucidation of the Isolates

**Figure 1:**
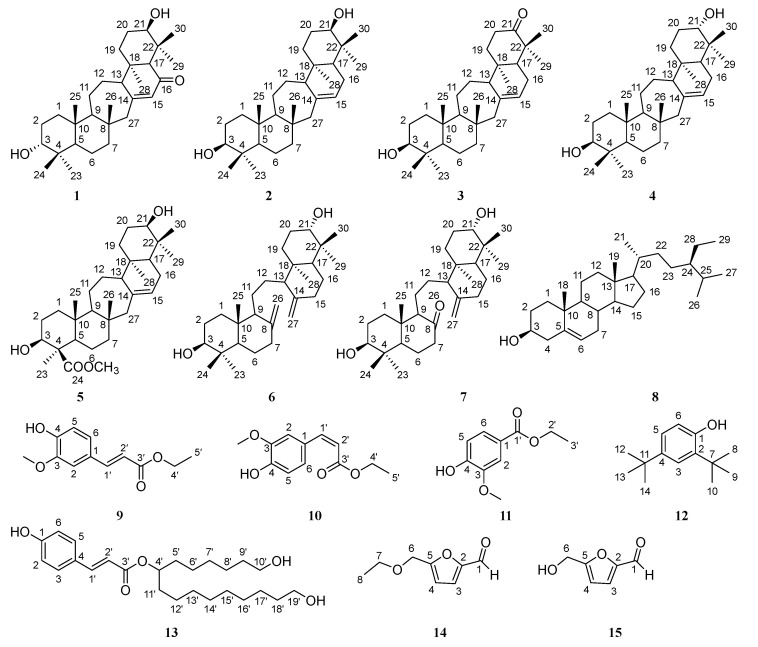
The chemical structures of the isolates **1**–**15** from *L. japonicum*.

A phytochemical investigation of the aerial parts of *L. japonicum* led to the isolation of fifteen structurally diverse compounds **1**–**15** (Figure 1). Their chemical structures were determined based on an analysis of nuclear magnetic resonance (NMR) data and a comparison with those reported in the literature (Table 1).

The ^1^H and ^13^C NMR spectra of chemical constituent **1** showed the signals attributable to one α,β-unsaturated ketone [δ_H_ 5.71 (1H, t, *J* = 2.5 Hz, H-15); δ_C_ 201.4 (C-16), 163.7 (C-14), 128.8 (C-15)], two oxygenated methines [δ_H_ 3.41 (1H, t, *J* = 2.9 Hz, H-3), 3.35 (1H, t, *J* = 2.9 Hz, H-21); δ_C_ 76.9 (C-3), 76.1 (C-21)], and seven methyls [δ_H_ 0.95 (3H, s, H-23), 0.87 (3H, s, H-24), 0.83 (3H, s, H-25), 0.80 (3H, s, H-26), 0.84 (3H, s, H-28), 1.12 (3H, s, H-29), 1.25 (3H, s, H-30); δ_C_ 28.5 (C-23), 22.3 (C-24), 15.8 (C-25), 20.1 (C-26), 15.0 (C-28), 21.6 (C-29), 28.0 (C-30)]. Considering the features revealed by the ^1^H and ^13^C NMR spectra, this compound likely belonged to the family of serratane triterpenes [5]. It was found that the overall ^1^H and ^13^C NMR spectral data of **1** displayed a high degree of resemblance to those of the serratane triterpene 16-oxo-3α-hydroxyserrat-14-en-21β-ol [16] (Table S1). Consequently, the structure of compound **1** was depicted as shown in Figure 1.

In the ^1^H and ^13^C NMR spectra of phytochemical **2**, the signals attributable to one double bond [δ_H_ 5.32 (1H, br s, H-15); δ_C_ 138.7 (C-14), 122.2 (C-15)], two oxygenated methines [δ_H_ 3.45 (1H, t, *J* = 2.9 Hz, H-21), 3.18 (1H, dd, *J* = 11.7, 4.6 Hz, H-3); δ_C_ 79.0 (C-3), 76.4 (C-21)], and seven methyls [δ_H_ 0.80 (3H, s, H-23), 0.88 (3H, s, H-24), 0.77 (3H, s, H-25), 0.84 (3H, s, H-26), 0.69 (3H, s, H-28), 0.93 (3H, s, H-29), 0.97 (3H, s, H-30); δ_C_ 28.3 (C-23), 15.6 (C-24), 15.9 (C-25), 19.9 (C-26), 13.4 (C-28), 21.9 (C-29), 27.9 (C-30)] were observed. These characteristic ^1^H and ^13^C NMR spectral data suggested that this compound was a serratane-type triterpene. The subsequent literature survey revealed the overall ^1^H and ^13^C NMR spectral data of **2** resembled those of 21-*epi*-serratenediol [24] (Table S2). Therefore, the structure of compound **2** was determined as displayed in Figure 1.

The ^1^H and ^13^C NMR spectral data of compound **3** indicated the presence of one double bond [δ_H_ 5.38 (1H, br s, H-15); δ_C_ 138.5 (C-14), 122.1 (C-15)], one carbonyl [δ_C_ 217.2 (C-21)], one oxygenated methine [δ_H_ 3.19 (1H, dd, *J* = 11.7, 4.6 Hz, H-3); δ_C_ 79.0 (C-3)], and seven methyls [δ_H_ 0.97 (3H, s, H-23), 0.77 (3H, s, H-24), 0.80 (3H, s, H-25), 0.83 (3H, s, H-26), 0.92 (3H, s, H-28), 1.04 (3H, s, H-29), 1.08 (3H, s, H-30); δ_C_ 28.3 (C-23), 15.6 (C-24), 15.9 (C-25), 19.9 (C-26), 13.1 (C-28), 24.6 (C-29), 21.7 (C-30)]. The ^1^H and ^13^C NMR spectra of compound **3** exhibited close similarity to those of **2**, except for the appearance of a carbonyl group in **3** instead of one oxygenated methine group in **2**. Accordingly, compound **3** was identified as 3β-hydroxy-14-serraten-21-one as depicted in Figure 1, whose data matched well with those recorded in the literature [25] (Table S3).

Interestingly, the ^1^H and ^13^C NMR spectral data of compound **4** exhibited close similarity to those of **2**, including one double bond [δ_H_ 5.33 (1H, br s, H-15); δ_C_ 138.3 (C-14), 122.3 (C-15)], two oxygenated methines [δ_H_ 3.23 (1H, dd, *J* = 11.6, 4.2 Hz, H-21), 3.19 (1H, dd, *J* = 11.7, 4.6 Hz, H-3); δ_C_ 79.0 (C-3), 79.3 (C-21)], and seven methyls [δ_H_ 0.80 (3H, s, H-23), 0.83 (6H, s, H-24, H-26), 0.77 (3H, s, H-25), 0.67 (3H, s, H-28), 0.96 (3H, s, H-29), 0.97 (3H, s, H-30); δ_C_ 15.6 (C-23), 28.3 (C-24), 15.9 (C-25), 19.9 (C-26), 13.6 (C-28), 14.8 (C-29), 27.7 (C-30)]. Their difference was in the ^1^H and ^13^C NMR data of the oxygenated methine at C-3, revealing the different configurations of the hydroxy group at C-3. Thus, **4** was identified as serratenediol (Figure 1), which was confirmed by the almost superposable ^1^H and ^13^C NMR data reported in the literature [24,26] (Table S4).

Inspection of the ^1^H and ^13^C NMR spectra of compound **5** revealed there was one double bond [δ_H_ 5.51 (1H, br s, H-15); δ_C_ 139.8 (C-14), 123.3 (C-15)], two oxygenated methines [δ_H_ 3.70 (1H, br s, H-21), 3.36 (1H, dd, *J* = 12.2, 4.6 Hz, H-3); δ_C_ 78.4 (C-3), 75.6 (C-21)], and six methyls [δ_H_ 1.58 (3H, s, H-23), 0.8 (3H, s, H-25), 0.88 (3H, s, H-26), 0.85 (3H, s, H-28), 0.97 (3H, s, H-29), 1.18 (3H, s, H-30); δ_C_ 24.6 (C-23), 14.1 (C-25), 20.1 (C-26), 14.2 (C-28), 22.5 (C-29), 29.1 (C-30)] in the chemical structure of **5**. As revealed by the ^1^H and ^13^C NMR data, **5** exhibited close structural similarity with **2**. However, the remarkably different ^1^H and ^13^C NMR data of one ester functionality [δ_H_ 3.64 (3H, s, -OCH_3_); *δ*_C_ 178.4 (C-24)] in **5** were observed, which indicated that the methyl group in **2** was replaced by the methyl ester group in **5**. Based on the above-mentioned analysis along with an extensive literature survey [27,28] (Table S5), compound **5** was identified as methyl lycernuate A, as displayed in Figure 1.

The ^1^H and ^13^C NMR spectra of chemical constituent **6** showed the signals attributable to two double bonds [δ_H_ 4.83 (2H, s, H-26a, H-27a), 4.56 (2H, s, H-26b, H-27b); δ_C_ 148.6 (C-8, C-14), 106.9 (C-26, C-27)], two oxygenated methines [δ_H_ 3.25 (2H, d, *J* = 11.7 Hz, H-3, H-21); δ_C_ 79.1 (C-3, C-21)], and six methyls [δ_H_ 0.99 (6H, s, H-23, H-30), 0.76 (6H, s, H-24, H-29), 0.64 (6H, s, H-25, H-28); δ_C_ 28.5 (C-23, C-29), 15.5 (C-24, C-30), 14.7 (C-25, C-28)]. These characteristic ^1^H and ^13^C NMR data indicated that this compound was a serratane-type triterpene [5]. A further literature survey revealed that the overall ^1^H and ^13^C NMR data of **6** displayed a high degree of resemblance to those of α-onocerin [29] (Table S6).

The ^1^H and ^13^C NMR spectra of **7** exhibited close similarity to those of **6**, including one double bond [δ_H_ 4.90 (2H, s, H-27); δ_C_ 147.5 (C-14), 108.0 (C-27)], two oxygenated methines [δ_H_ 3.33 (1H, d, *J* = 11.6 Hz, H-3), 3.23 (1H, d, *J* = 11.6 Hz, H-21); δ_C_ 78.8 (C-3), 79.1 (C-21)], and six singlet methyls [δ_H_ 0.69 (3H, s, H-23), 0.81 (3H, s, H-24), 1.09 (3H, s, H-25), 0.99 (3H, s, H-28), 0.63 (3H, s, H-29), 0.76 (3H, s, H-30); δ_C_ 27.7 (C-23), 15.5 (C-24), 14.6 (C-25), 14.9 (C-28), 28.5 (C-29), 15.5 (C-30)]. Their difference was in the appearance of a carbonyl [δ_C_ 212.1 (C-8)] in **7** instead of the one double bond [δ_H_ 4.83 (1H, s, H-26a), 4.56 (1H, s, H-26b); δ_C_ 148.6 (C-8), 106.9 (C-26)] in **6**. Accordingly, compound **7** was identified as 26-nor-8-oxo-α-onocerin, whose data matched well with those recorded in the literature [30] (Table S7).

In the ^1^H and ^13^C NMR spectra of compound **8**, one double bond [δ_H_ 5.35 (1H, m); δ_C_ 140.9 (s), 121.9 (d)], one oxygenated methine [δ_H_ 3.52 (1H, m); δ_C_ 72.0 (d)], and six methyls [δ_H_ 1.01 (3H, s), 0.92 (3H, d, *J* = 6.6 Hz), 0.84 (3H, t, *J* = 7.7 Hz), 0.83 (3H, d, *J* = 6.7 Hz), 0.81 (3H, d, *J* = 6.8 Hz), 0.68 (3H, s); δ_C_ 12.0 (q), 19.2 (q), 18.9 (q), 19.5 (q), 20.0 (q), 12.1 (q)] were recognized. It was found that the ^1^H and ^13^C NMR spectral data of **8** were identical to those of the *β*-stiosterol [23] (Table S8). Consequently, the structure of chemical composition **8** was assigned as displayed in Figure 1.

In the ^1^H and ^13^C NMR spectra of compound **9**, one α,β-conjugated carbonyl [δ_H_ 7.61 (1H, d, *J* = 15.9 Hz, H-1′), 6.29 (1H, d, *J* = 15.9 Hz, H-2′); δ_C_ 144.8 (C-1′), 115.8 (C-2′), 167.4 (C-3′)], one tri-substituted benzene [δ_H_ 7.03 (1H, d, *J* = 2.0 Hz, H-2), 6.92 (1H, d, *J* = 8.2 Hz, H-5), 7.07 (1H, dd, *J* = 8.2, 1.9 Hz, H-6); δ_C_ 127.2 (C-1), 109.4 (C-2), 148.0 (C-3), 146.9 (C-4), 114.8 (C-5), 123.2 (C-6)], one methoxyl [δ_H_ 3.93 (3H, s, -OCH_3_); δ_C_ 56.1 (-OCH_3_)], and one ethoxyl [δ_H_ 4.26 (2H, q, *J* = 7.1 Hz, H-4′), 1.33 (3H, t, *J* =7.1 Hz, H-5′); δ_C_ 60.5 (C-4′), 14.5 (C-5′)] were observed. These NMR data revealed that the chemical structure of compound **9** was *trans*-ethyl ferulate, which was confirmed by the well-matched data in the literature [31] (Table S9).

The ^1^H and ^13^C NMR spectra of compound **10** showed the signals attributed to one α,β-conjugated carbonyl [δ_H_ 6.79 (1H, d, *J* = 12.9 Hz, H-1′), 5.81 (1H, d, *J* = 12.9 Hz, H-2′); δ_C_ 143.8 (C-1′), 117.0 (C-2′), 166.7 (C-3′)], one tri-substituted benzene [δ_H_ 7.77 (1H, d, *J* = 2.0 Hz, H-2), 6.88 (1H, d, *J* = 8.2 Hz, H-5), 7.11 (1H, dd, *J* = 8.3, 2.0 Hz, H-6); δ_C_ 127.4 (C-1), 112.9 (C-2), 146.0 (C-3), 147.2 (C-4), 113.9 (C-5), 125.8 (C-6)], one methoxyl [δ_H_ 3.93 (3H, s, -OCH_3_); δ_C_ 56.1 (-OCH_3_)], and one ethoxyl [δ_H_ 4.19 (2H, q, *J* = 7.1 Hz, H-4′), 1.29 (3H, t, *J* = 7.2 Hz, H-5′); δ_C_ 60.3 (C-4′), 14.4 (C-5′)]. These data indicated the close structural similarity between **10** and **9**. However, the smaller coupling constant 12.0 Hz between H-1′ and H-2′ suggested the *cis*-geometry of the double bond Δ^1′^. Thus, **10** was identified as *cis*-ethyl ferulate, which was supported by the comparison of the data reported in the literature [31,32] (Table S10).

As shown in the ^1^H and ^13^C NMR spectra of compound **11**, one carbonyl carbon [δ_C_ 166.6 (C-1′)], one tri-substituted benzene [δ_H_ 7.55 (1H, d, *J* = 1.9 Hz, H-2), 6.93 (1H, d, *J* = 8.3 Hz, H-5), 7.64 (1H, dd, *J* = 8.3, 1.9 Hz, H-6); δ_C_ 124.2 (C-1), 111.8 (C-2), 146.3 (C-3), 150.1 (C-4), 114.1 (C-5), 122.8 (C-6)], one methoxyl [δ_H_ 3.95 (3H, s, -OCH_3_); δ_C_ 56.2 (-OCH_3_)], and one ethoxyl [δ_H_ 4.35 (2H, q, *J* =7.1 Hz, H-2′), 1.38 (3H, t, *J* = 7.1 Hz, H-3′); δ_C_ 60.9 (C-2′), 14.5 (C-3′)] were recognized. It was found that the ^1^H and ^13^C NMR spectral data of **11** were identical to those of ethyl 4-hydroxy-3-methoxybenzoate [33] (Table S11), which are displayed in Figure 1.

In the ^1^H and ^13^C NMR spectra of compound **12**, the signals attributed to one tri-substituted benzene [δ_H_ 7.31 (1H, d, *J* = 2.4 Hz, H-3), 7.08 (1H, dd, *J* = 8.2, 2.4 Hz, H-5), 6.60 (1H, d, *J* = 8.2 Hz, H-6); (δ_C_ 151.9 (C-1), 143.1 (C-2), 116.1 (C-3), 135.3 (C-4), 123.7 (C-5), 124.2 (C-6)], and six methyls [δ_H_ 1.30 (9H, s, H-8, H-9, H-10), 1.43 (9H, s, H-12, H-13, H-14); δ_C_ 31.8 (C-8, C-9, C-10), 29.8 (C-12, C-13, C-14)] were observed. It was revealed that the ^1^H and ^13^C NMR spectral data of **12** resembled those of the 2,4-di-*tert*-butylphenol [34] (Table S12). As a result, the structure of chemical composition **12** was determined as shown in Figure 1.

In the ^1^H and ^13^C NMR spectra of compound **13**, one α,β-conjugated carbonyl [δ_H_ 7.61 (1H, d, *J* = 15.9 Hz, H-1′), 6.33 (1H, d, *J* = 15.9 Hz, H-2′); δ_C_ 146.3 (C-1′), 115.6 (C-2′), 169.3 (C-3′)], and one *para*-disubstituted benzene [δ_H_ 6.82 (2H, d, *J* = 8.6, H-2, H-6), 7.48 (2H, d, *J* = 8.6, H-3, H-5); δ_C_ 161.3 (C-1), 116.8 (C-2), 131.1 (C-3), 127.2 (C-4), 131.1 (C-5), 116.8 (C-6)] were observed. It was found that the ^1^H and ^13^C NMR spectral data of **13** were identical to those of the ginkwanghol A [35] (Table S13). Consequently, the structure of chemical composition **13** was assigned as displayed in Figure 1.

As shown in the ^1^H and ^13^C NMR spectra of compound **14**, the presence of one aldehyde [δ_H_ 9.62 (1H, s, -CHO); δ_C_ 177.9 (C-1)], one di-substituted furan ring [δ_H_ 6.52 (1H, d, *J* = 3.6 Hz, H-3), 7.21 (1H, d, *J* = 3.5 Hz, H-4); δ_C_ 158.9 (C-2), 122.1 (C-3), 111.1 (C-4), 152.7 (C-5)], one oxymethylene [δ_H_ 4.53 (2H, s, H-6); δ_C_ 66.8 (C-6)], and one ethoxyl [δ_H_ 3.59 (2H, q, *J* = 7.0 Hz, H-7), 1.24 (3H, t, *J* = 7.0 Hz, H-8); δ_C_ 64.9 (C-7), 15.2 (C-8)] was recognized. The literature survey revealed that the overall ^1^H and ^13^C NMR spectra data of **14** were identical to those reported for 5-ethoxymethyl furfural [36] (Table S14).

In the ^1^H and ^13^C NMR spectra of compound **15**, the signals attributed to one aldehyde carbon [δ_H_ 9.60 (1H, s, -CHO); δ_C_ 177.8 (C-1)], one di-substituted furan ring [δ_H_ 6.52 (1H, d, *J* = 3.5 Hz, H-3), 7.21 (1H, d, *J* = 3.5 Hz, H-4); δ_C_ 160.6 (C-2), 122.3 (C-3), 110.1 (C-4), 152.6 (C-5)], and one hydroxymethyl [δ_H_ 4.72 (2H, s, H-6); δ_C_ 57.8 (C-6)] were observed. It was revealed that the overall ^1^H and ^13^C NMR spectra data of **15** resembled those of 5-hydroxymethyl furfural [37] (Table S15). Consequently, the structure of compound **15** was assigned as shown in Figure 1.

Among these isolated compounds, **1**, **2**, **4**, **6**, **7**, and **8** had been reported from the species *L*. *japonicum* [12,13,16,24,38,39,40] in these earlier investigations, respectively. In other words, the other nine compounds were first found in the species *L*. *japonicum*.

### 2.2. Tyrosinase Inhibitory Bioassays

The tyrosinase inhibitory effects of substances **6**–**9** were evaluated in our previous studies [23,41]. In this study, all of the other isolates were subjected to tyrosinase inhibitory bioassay. As a result (Table 2), compounds **4**, **12**, **13**, **14,** and **15** exhibited different levels of tyrosinase inhibition, with IC_50_ values differing from 1.5 mM to 6.8 mM.

### 2.3. Molecular Docking Studies

**Figure 2:**
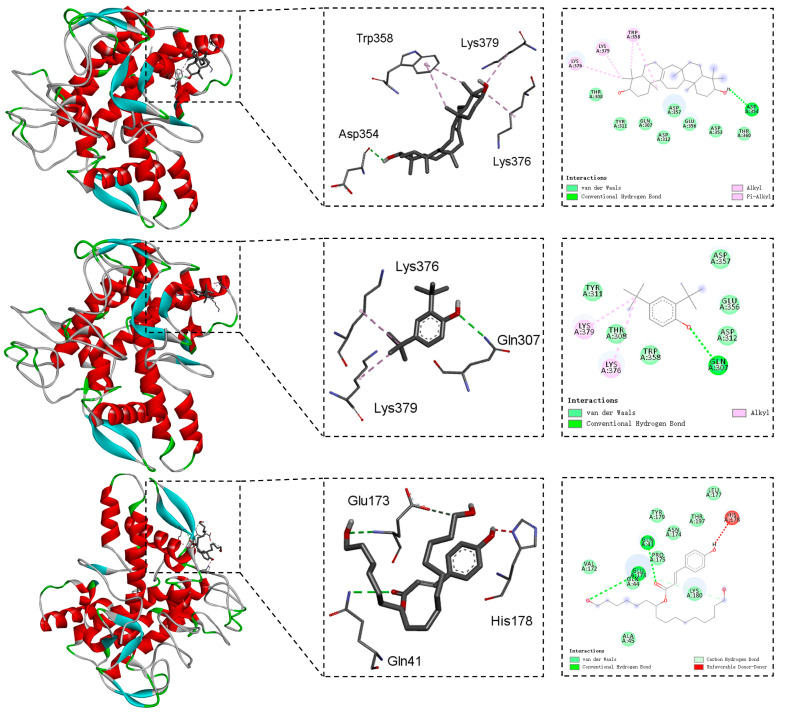
The binding modes between compounds **4**, **12**, and **13** and the mushroom tyrosinase (PDB: 2Y9X).

**Figure 3:**
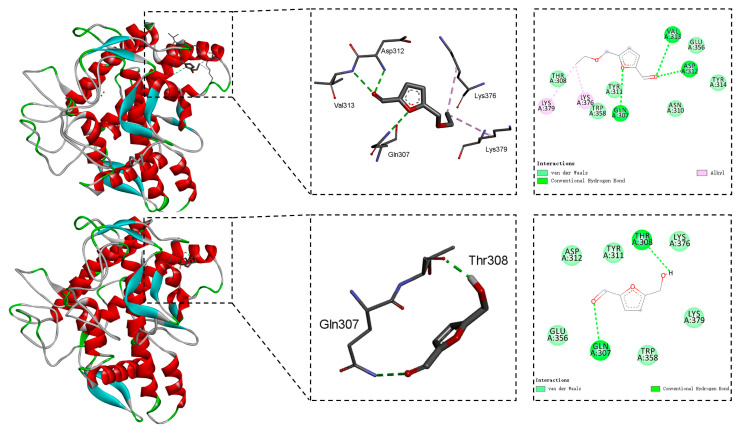
The binding modes between compounds **14** and **15** and the mushroom tyrosinase (PDB: 2Y9X).

The binding mechanisms between the five components, **4**, **12**, **13**, **14**, and **15,** and the mushroom tyrosinase were deciphered through molecular docking calculations [42]. Figure 2 and Figure 3 represent the 2D and 3D interactive plots of these constituents with tyrosinase in the docked complexes.

As for compound **4**, its hydroxyl group formed one hydrogen bond with the amino acid Asp354. Additionally, two alkyl interactions with Lys376, and Lys379 and one alkyl stacking interaction with Trp358 were observed (Figure 2). Figure 2 also illustrates that the hydrogen bond with amino acid Gln307 and alkyl stacking interactions with Lys376 and Lys379 were engaged in the binding mode between constituent **12** and the mushroom tyrosinase. Figure 2 also demonstrates the different types of interactions in the complexes of compound **13** and the mushroom tyrosinase. One hydrogen bond was formed between the carbonyl at C-3′ of **13** and amino acid residue Gln41, while another hydrogen bond was established by the hydroxyl groups at C-10′ and Glu173. Furthermore, the hydroxyl groups at C-19′ of substance **13** engaged in a carbon hydrogen bond with Lys180.

As shown in Figure 3, the hydrogen bonds and alkyl interactions played key roles in the binding mode between component **14** and the mushroom tyrosinase. The amino acids Lys376 and Lys379 were involved in the alkyl interactions [43]. Furthermore, substance **14** induced hydrogen bonds with Gln307, Val313, and Asp312. Figure 3 also disclosed that compound **15** formed two hydrogen bonds with the amino acids Gln307 and Thr308.

## 3. Materials and Methods

### 3.1. General Experimental Procedures

NMR spectra were measured on Bruker DRX-400 and 600 spectrometers (Bruker Biospin AG, Fällanden, Germany). Commercial silica gel (200–300 and 300–400 mesh, Qingdao Haiyang Chemical Group Co., Ltd., Qingdao, China) and Sephadex LH-20 gel (Amersham Biosciences, Amersham, UK) were used for column chromatography, and precoated silica gel plates (GF-254, Yan Tai Zi Fu Chemical Group Co., Yantai, China) were used for analytical TLC. All solvents used for column chromatography were of analytical grade (Shanghai Chemical Reagents Co., Ltd., Shanghai, China).

### 3.2. Plant Material

The aboveground parts of the plant *L. japonicum* were collected from Baiyunxian Mountain in the Hunan Province of China in January 2023. The authentication of the plant sample was performed by Dr. L. Wu, one author of this manuscript. A specimen (No. P2023HN2) for certification was kept in the Laboratory of Natural Products Chemistry, Central South University of Forestry and Technology (Changsha, China).

### 3.3. Extraction and Isolation

The aerial parts of the plant *L. japonicum* were air-dried (dried weight 2.2 kg). They were extracted with 90% ethanol using ultrasonication at room temperature (2 h × 3). A dark residue was obtained after the removal of the solvent from the combined ethanol extracts by a rotavapor. Then, the suspension of the residue in H_2_O was successively partitioned with the solvents petroleum ether (P) and ethyl acetate (E) to yield two corresponding P and E extracts, respectively.

The E extract was first chromatographed over silica gel (P/E 20:1→0:1) to give seven fractions, Fr. A–G. Six subfractions, Fr. C1–C6, were obtained from Fr. C by silica gel column chromatography (P/E 20:1→1:1). Fr. C2 was further divided into three subfractions (Fr. C2A–C2C) by chromatography over silica gel (P/E 20:1→5:1). Purification of Fr. C2A by chromatography over silica gel (P/E 20:1) yielded compound **12** (9.5 mg). Similarly, purification of Fr. C3 afforded component **9** (15.3 mg) using silica gel column chromatography (P/E 10:1→3:1). Separation of Fr. C4 by silica gel (dichromethane (D)/methanol (M) 200:1→50:1) yielded five subfractions, Fr. C4A–C4F. Purification of Fr. C4B by silica gel (P/E 7:1→3:1) resulted in three subfractions, Fr. C4B1–C4B3. Compound **14** (5.9 mg) was obtained from Fr. C4B2 by chromatography over Sephadex LH-20 (P/D/M 2:1:1). Compound **8** (38.3 mg) was obtained from Fr. C4B3 by silica gel column chromatography (P/E 7:1). Three subfractions, Fr. C4C1–C4C3, were revealed by silica gel column chromatography of Fr. C4C (D/M 200:1→100:1). Compound **11** (4.1 mg) was obtained from Fr. C4C2 by chromatography over Sephadex LH-20 (P/D/M 2:1:1). Separation of Fr. C4D by silica gel (P/E 5:1→3:1) gave four subfractions, Fr. C4D1–C4D4. Compound **1** (2.2 mg) was isolated from Fr. C4D3 by silica gel column chromatography (P/E 4:1). Fr. C5 was divided into six subfractions, Fr. C5A–C5F by chromatography over silica gel (P/E 5:1→3:1). Compound **2** (14.3 mg) was obtained from Fr. C5C by silica gel column chromatography (D/M 100:1). Fr. C5E (35.2 mg) was successively processed using chromatography over silica gel (D/M 100:1→30:1) and Sephadex LH-20 (P/D/M 2:1:1) to produce compound **3** (7.9 mg).

Fr. F was divided into five subfractions, Fr. F1–F5, by silica gel column chromatography (D/M 100:1→5:1). Compound **6** (573.9 mg) was isolated from Fr. F1 by chromatography over silica gel (P/E 3:1). Fr. F2 was divided into three subfractions, Fr. F2A–F2C by silica gel column chromatography (P/E 3:1→1:1). Compound **10** (8.7 mg) was obtained from Fr. F2B by chromatography over silica gel (D/M 100:1). Purification of F2C by silica gel column chromatography (D/M 80:1) produced compound **4** (13.1 mg). Separation of Fr. F3 by silica gel column chromatography (D/M 50:1→20:1) yielded five subfractions, Fr. F3A–F3E. Compound **7** (4.8 mg) was obtained from Fr. F3B by chromatography over silica gel (P/E 7:3). Compound **5** (7.4 mg) was isolated from Fr. F3D by silica gel column chromatography (P/E 2:1). Fr. F4 (176.4 mg) was purified by silica gel column chromatography (P/E 2:1→1:1) to give four subfractions, Fr. F4A–F4D. Purification of Fr. F4B by chromatography over Sephadex LH-20 (P/D/M 2:1:1) resulted in three subfractions, Fr. F4B1–F4B3. Compound **15** (12.4 mg) was obtained from Fr. F4B3 by silica gel column chromatography (D/M 20:1). Fr. F5 (428.2 mg) was divided into six subfractions, Fr. F5A–F5F by chromatography over silica gel (P/E 2:1→0:1) Separation of Fr. F5D by chromatography over Sephadex LH-20 CC (P/D/M 2:1:1) yielded two subfractions, Fr. F5D1–F5D2. Compound **13** (10.6 mg) was isolated from Fr. F5D2 using silica gel column chromatography (P/E 1:1).

**Figure 4:**
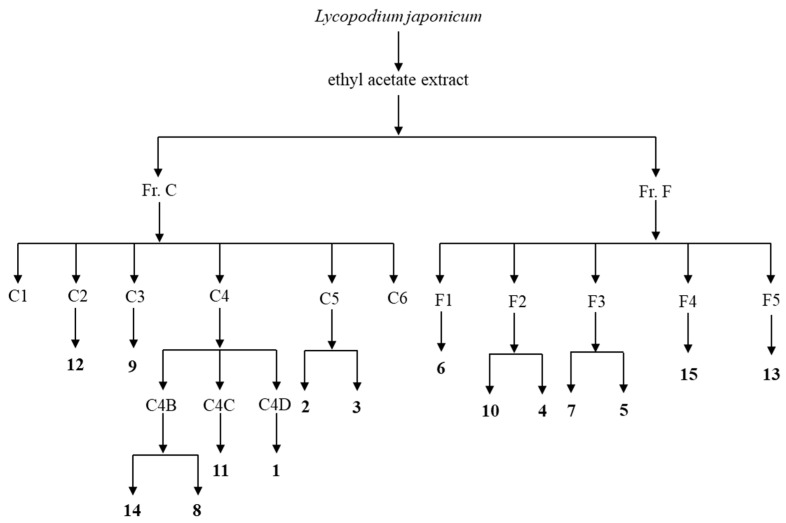
The summary of key experimental procedures of compounds **1**–**15**.

Figure 4 summarizes the aforementioned key isolation and purification experimental procedures of components **1**–**15**.

### 3.4. Characteristic ^1^H and ^13^C NMR Spectral Data of Isolates

Compound **1**: ^1^H-NMR (600 MHz, CDCl_3_) δ: 5.71 (1H, t, *J* = 2.5 Hz, H-15), 3.41 (1H, t, *J* = 2.9 Hz, H-3), 3.35 (1H, t, *J* = 2.9 Hz, H-21), 1.25 (3H, s, H-30), 1.12 (3H, s, H-29), 0.95 (3H, s, H-23), 0.87 (3H, s, H-24), 0.84 (3H, s, H-28), 0.83 (3H, s, H-25), 0.80 (3H, s, H-26); ^13^C-NMR (150 MHz, CDCl_3_) δ: 33.3 (C-1), 26.6 (C-2), 76.9 (C-3), 38.4 (C-4), 49.4 (C-5), 18.8 (C-6), 45.0 (C-7), 38.2 (C-8), 62.6 (C-9), 37.8 (C-10), 24.7 (C-11), 25.6 (C-12), 59.0 (C-13), 163.7 (C-14), 128.8 (C-15), 201.4 (C-16), 58.8 (C-17), 44.4 (C-18), 31.6 (C-19), 25.2 (C-20), 76.1 (C-21), 36.9 (C-22), 28.5 (C-23), 22.3 (C-24), 15.8 (C-25), 20.1 (C-26), 56.0 (C-27), 15.0 (C-28), 21.6 (C-29), 28.0 (C-30).

Compound **2**: ^1^H-NMR (600 MHz, CDCl_3_) δ: 5.32 (1H, br s, H-15), 3.45 (1H, t, *J* = 2.9 Hz, H-21), 3.18 (1H, dd, *J* = 11.7, 4.6 Hz, H-3), 0.97 (3H, s, H-30), 0.93 (3H, s, H-29), 0.88 (3H, s, H-24), 0.84 (3H, s, H-26), 0.80 (3H, s, H-23), 0.77 (3H, s, H-25), 0.69 (3H, s, H-28); ^13^C-NMR (150 MHz, CDCl_3_) δ: 38.8 (C-1), 27.3 (C-2), 79.0 (C-3), 38.3 (C-4), 55.9 (C-5), 19.1 (C-6), 45.3 (C-7), 37.6 (C-8), 63.1 (C-9), 39.1 (C-10), 25.4 (C-11), 27.7 (C-12), 57.0 (C-13), 138.7 (C-14), 122.2 (C-15), 24.2 (C-16), 43.5 (C-17), 36.1 (C-18), 31.4 (C-19), 25.6 (C-20), 76.4 (C-21), 37.3 (C-22), 28.3 (C-23), 15.6 (C-24), 15.9 (C-25), 19.9 (C-26), 56.4 (C-27), 13.4 (C-28), 21.9 (C-29), 27.9 (C-30).

Compound **3**: ^1^H-NMR (600 MHz, CDCl_3_) δ: 5.38 (1H, br s, H-15), 3.19 (1H, dd, *J* = 11.7, 4.6 Hz, H-3), 1.08 (3H, s, H-30), 1.04 (3H, s, H-29), 0.97 (3H, s, H-23), 0.92 (3H, s, H-28), 0.83 (3H, s, H-26), 0.80 (3H, s, H-25), 0.77 (3H, s, H-24); ^13^C-NMR (150 MHz, CDCl_3_) δ: 38.7 (C-1), 27.7 (C-2), 79.0 (C-3), 38.3 (C-4), 51.3 (C-5), 19.0 (C-6), 45.3 (C-7), 37.2 (C-8), 62.9 (C-9), 39.1 (C-10), 25.6 (C-11), 27.3 (C-12), 56.6 (C-13), 138.5 (C-14), 122.1 (C-15), 24.7 (C-16), 55.9 (C-17), 36.3 (C-18), 34.9 (C-19), 38.5 (C-20), 217.2 (C-21), 47.8 (C-22), 28.3 (C-23), 15.6 (C-24), 15.9 (C-25), 19.9 (C-26), 56.0 (C-27), 13.1 (C-28), 24.6 (C-29), 21.7 (C-30).

Compound **4**: ^1^H-NMR (600 MHz, CDCl_3_) δ: 5.33 (1H, br s, H-15), 3.23 (1H, dd, *J* = 11.6, 4.2 Hz, H-21), 3.19 (1H, dd, *J* = 11.7, 4.6 Hz, H-3), 0.97 (3H, s, H-30), 0.96 (3H, s, H-29), 0.83 (6H, s, H-24, H-26), 0.80 (3H, s, H-23), 0.77 (3H, s, H-25), 0.67 (3H, s, H-28); ^13^C-NMR (150 MHz, CDCl_3_) δ: 38.7 (C-1), 27.8 (C-2), 79.0 (C-3), 39.0 (C-4), 55.8 (C-5), 19.0 (C-6), 45.3 (C-7), 37.2 (C-8), 63.0 (C-9), 38.3 (C-10), 25.4 (C-11), 27.4 (C-12), 57.3 (C-13), 138.3 (C-14), 122.3 (C-15), 24.2 (C-16), 49.6 (C-17), 36.3 (C-18), 37.3 (C-19), 27.7 (C-20), 79.3 (C-21), 39.1 (C-22), 15.6 (C-23), 28.3 (C-24), 15.9 (C-25), 19.9 (C-26), 56.2 (C-27), 13.6 (C-28), 14.8 (C-29), 27.7 (C-30).

Compound **5**: ^1^H-NMR (600 MHz, C_5_D_5_N) δ: 5.51 (1H, br s, H-15), 3.70 (1H, br s, H-21), 3.64 (3H, s, -OCH_3_), 3.36 (1H, dd, *J* = 12.2, 4.6 Hz, H-3), 1.58 (3H, s, H-23), 1.18 (3H, s, H-30), 0.97 (3H, s, H-29), 0.88 (3H, s, H-26), 0.85 (3H, s, H-28), 0.80 (3H, s, H-25); ^13^C-NMR (150 MHz, C_5_D_5_N) δ: 39.8 (C-1), 29.3 (C-2), 78.4 (C-3), 50.2 (C-4), 57.0 (C-5), 21.4 (C-6), 45.7 (C-7), 37.6 (C-8), 62.9 (C-9), 39.0 (C-10), 27.0 (C-11), 28.1 (C-12), 57.7 (C-13), 139.8 (C-14), 123.3 (C-15), 25.0 (C-16), 44.2 (C-17), 36.8 (C-18), 32.2 (C-19), 26.0 (C-20), 75.6 (C-21), 38.4 (C-22), 24.6 (C-23), 178.4 (C-24), 14.1 (C-25), 20.1 (C-26), 57.2 (C-27), 14.2 (C-28), 22.5 (C-29), 29.1 (C-30), 51.4 (-OCH_3_).

Compound **6**: ^1^H-NMR (600 MHz, CDCl_3_) δ: 4.83 (2H, s, H-26a, H-27a), 4.56 (2H, s, H-26b, H-27b), 3.25 (2H, dd, *J* = 11.7, 4.2 Hz, H-3, H-21), 0.99 (6H, s, H-23, H-30), 0.76 (6H, s, H-24, H-29), 0.64 (6H, s, H-25, H-28); ^13^C-NMR (150 MHz, CDCl_3_) δ: 37.2 (C-1, C-19), 28.1 (C-2, C-20), 79.1 (C-3, C-21), 39.4 (C-4, C-22), 54.8 (C-5, C-17), 24.2 (C-6, C-16), 38.4 (C-7, C-15), 148.6 (C-8, C-14), 57.7 (C-9, C-13), 39.3 (C-10, C-18), 22.8 (C-11, C-12), 28.5 (C-23, C-29), 15.5 (C-24, C-30), 14.7 (C-25, C-28), 106.9 (C-26, C-27).

Compound **7**: ^1^H-NMR (400 MHz, CDCl_3_) δ: 4.91 (1H, s, H-27a), 4.89 (1H, s, H-27b), 3.34 (1H, dd, *J* = 11.7, 4.0 Hz, H-3), 3.23 (1H, dd, *J* = 11.8, 4.2 Hz, H-21), 1.09 (3H, s, H-25), 0.99 (3H, s, H-28), 0.81 (3H, s, H-24), 0.76 (3H, s, H-30), 0.69 (3H, s, H-23), 0.63 (3H, s, H-29); ^13^C-NMR (100 MHz, CDCl_3_) δ: 37.1 (C-1), 28.5 (C-2), 78.8 (C-3), 39.3 (C-4), 54.8 (C-5), 27.7 (C-6), 42.5 (C-7), 212.1 (C-8), 64.8 (C-9), 42.4 (C-10), 21.2 (C-11), 23.7 (C-12), 57.4 (C-13), 147.5 (C-14), 37.3 (C-15), 23.7 (C-16), 53.7 (C-17), 38.4 (C-18), 37.1 (C-19), 28.1 (C-20), 79.1 (C-21), 39.4 (C-22), 27.7 (C-23), 15.5 (C-24), 14.6 (C-25), 108.0 (C-27), 14.9 (C-28), 28.5 (C-29), 15.5 (C-30).

Compound **8**: ^1^H-NMR (600 MHz, CDCl_3_) δ: 5.35 (1H, m, H-6), 3.52 (1H, m, H-3), 1.01 (3H, s, H-19), 0.92 (3H, d, *J* = 6.6 Hz, H-21), 0.84 (3H, t, *J* = 7.7 Hz, H-29), 0.83 (3H, d, *J* = 6.7 Hz, H-26), 0.81 (3H, d, *J* = 6.8 Hz, H-27), 0.68 (3H, s, H-18); ^13^C-NMR (150 MHz, CDCl_3_) δ: 37.4 (C-1), 32.1 (C-2), 72.0 (C-3), 42.4 (C-4), 140.9 (C-5), 121.9 (C-6), 32.1 (C-7), 31.8 (C-8), 50.3 (C-9), 36.7 (C-10), 21.2 (C-11), 39.9 (C-12), 42.5 (C-13), 56.9 (C-14), 24.5 (C-15), 28.4 (C-16), 56.2 (C-17), 12.0 (C-18), 19.2 (C-19), 36.3 (C-20), 18.9 (C-21), 34.1 (C-22), 26.2 (C-23), 46.0 (C-24), 29.3 (C-25), 19.5 (C-26), 20.0 (C-27), 23.2 (C-28), 12.1 (C-29).

Compound **9**: ^1^H-NMR (600 MHz, CDCl_3_) δ: 7.61 (1H, d, *J* = 15.9 Hz, H-1′), 7.07 (1H, dd, *J* = 8.2, 2.0 Hz, H-6), 7.03 (1H, d, *J* = 1.9 Hz, H-2), 6.92 (1H, d, *J* = 8.2 Hz, H-5), 6.29 (1H, d, *J* = 15.9 Hz, H-2′), 5.84 (1H, br s, 4-OH), 4.26 (2H, q, *J* = 7.1 Hz, H-4′), 3.93 (3H, s, -OCH_3_), 1.33 (3H, t, *J* =7.1 Hz, H-5′); ^13^C-NMR δ: (150 MHz, CDCl_3_) δ: 127.2 (C-1), 109.4 (C-2), 148.0 (C-3), 146.9 (C-4), 114.8 (C-5), 123.2 (C-6), 144.8 (C-1′), 115.8 (C-2′), 167.4 (C-3′), 60.5 (C-4′), 14.5 (C-5′), 56.1 (-OCH_3_).

Compound **10**: ^1^H-NMR (600 MHz, CDCl_3_) δ: 7.77 (1H, d, *J* = 2.0 Hz, H-2), 7.11 (1H, dd, *J* = 8.3, 2.0 Hz, H-6), 6.88 (1H, d, *J* = 8.2 Hz, H-5), 6.79 (1H, d, *J* = 12.9 Hz, H-1′), 5.82 (1H, br s, -OH), 5.81 (1H, d, *J* = 12.9 Hz, H-2′), 4.19 (2H, q, *J* = 7.1 Hz, H-4′), 3.93 (3H, s, -OCH_3_), 1.29 (3H, t, *J* = 7.2 Hz, H-5′); ^13^C-NMR (150 MHz, CDCl_3_) δ: 127.4 (C-1), 112.9 (C-2), 146.0 (C-3), 147.2 (C-4), 113.9 (C-5), 125.8 (C-6), 143.8 (C-1′), 117.0 (C-2′), 166.7 (C-3′), 60.3 (C-4′), 14.4 (C-5′), 56.1 (-OCH_3_).

Compound **11**: ^1^H-NMR (600 MHz, CDCl_3_) δ: 7.64 (1H, dd, *J* =8.3, 1.9 Hz, H-6), 7.55 (1H, d, *J* =1.9 Hz, H-2), 6.93 (1H, d, *J* =8.3 Hz, H-5), 4.35 (2H, q, *J* =7.1 Hz, H-2′), 3.95 (3H, s, -OCH_3_), 1.38 (3H, t, *J* = 7.1 Hz, H-3′); ^13^C-NMR (150 MHz, CDCl_3_) δ: 124.2 (C-1), 111.8 (C-2), 146.3 (C-3), 150.1 (C-4), 114.1 (C-5), 122.8 (C-6), 166.6 (C-1′), 60.9 (C-2′), 14.5 (C-3′), 56.2 (-OCH_3_).

Compound **12**: ^1^H-NMR (600 MHz, CDCl_3_) δ: 7.31 (1H, d, *J* = 2.4 Hz, H-3), 7.08 (1H, dd, *J* = 8.2, 2.4 Hz, H-5), 6.60 (1H, d, *J* = 8.2 Hz, H-6), 1.43 (9H, s, H-12, H-13, H-14), 1.30 (9H, s, H-8, H-9, H-10); ^13^C-NMR (150 MHz, CDCl_3_) δ: 151.9 (C-1), 143.1 (C-2), 116.1 (C-3), 135.3 (C-4), 123.7 (C-5), 124.2 (C-6), 34.4 (C-7), 31.8 (C-8, C-9, C-10), 34.9 (C-11), 29.8 (C-12, C-13, C-14).

Compound **13**: ^1^H-NMR (600 MHz, CD_3_OD) δ: 7.61 (1H, d, *J* =15.9 Hz, H-1′), 7.48 (2H, d, *J* = 8.6 Hz, H-3, H-5), 6.82 (2H, d, *J* = 8.6 Hz, H-2, H-6), 6.33 (1H, d, *J* = 15.9 Hz, H-2′), 5.01 (1H, m, H-4′), 3.55 (2H, t, *J* = 6.6 Hz, H-10′), 3.54 (2H, t, *J* = 6.6 Hz, H-19′), 1.63 (4H, m, H-5′, H-11′), 1.53 (4H, m, H-9′, H-18′); ^13^C-NMR (150 MHz, CD_3_OD) δ: 161.3 (C-1), 116.8 (C-2, C-6), 131.1 (C-3, C-5), 127.2 (C-4), 146.3 (C-1′), 115.6 (C-2′), 169.3 (C-3′), 75.4 (C-4′), 35.4 (C-5′, C-11′), 26.5 (C-6′), 30.7 (C-7′), 26.9 (C-8′), 33.7 (C-9′), 63.0 (C-10′), 26.4 (C-12′), 30.6 (C-13′, C-14′, C-15′), 30.5 (C-16′), 26.8 (C-17′), 33.5 (C-18′), 62.9 (C-19′).

Compound **14**: ^1^H-NMR (600 MHz, CDCl_3_) δ: 9.62 (1H, s, -CHO), 7.21 (1H, d, *J* = 3.5 Hz, H-4), 6.52 (1H, d, *J* = 3.6 Hz, H-3), 4.53 (2H, s, H-6), 3.59 (2H, q, *J* = 7.0 Hz, H-7), 1.24 (3H, t, *J* = 7.0 Hz, H-8); ^13^C-NMR (150 MHz, CDCl_3_) δ: 177.9 (C-1), 158.9 (C-2), 122.1 (C-3), 111.1 (C-4), 152.7 (C-5), 66.8 (C-6), 64.9 (C-7), 15.2 (C-8).

Compound **15**: ^1^H-NMR (600 MHz, CDCl_3_) δ: 9.60 (1H, s, -CHO), 7.21 (1H, d, *J* = 3.5 Hz, H-4), 6.52 (1H, d, *J* = 3.5 Hz, H-3), 4.72 (2H, s, H-6); ^13^C-NMR (150 MHz, CDCl_3_) δ: 177.8 (C-1), 160.6 (C-2), 122.3 (C-3), 110.1 (C-4), 152.6 (C-5), 57.8 (C-6).

### 3.5. In Vitro Tyrosinase Inhibitory Bioassay

The tyrosinase inhibition bioassay was conducted with minor modifications based on the previously described method [44]. In the wells of a 96-well plate, there was phosphate buffer (pH 6.8, 50 μL), tyrosinase (100 U/mL, 25 μL), sample solutions (25 μL), and L-tyrosine (100 μL). These mixtures were incubated at 37 °C for 0.5 h. Notably, 475 nm was selected for the measurement of absorbance of each well. The positive control was Kojic acid. The equation for the calculation of the inhibition rate was as follows: inhibition rate (%) = [1 − (A_1_ − A_2_)/(A_3_ − A_0_)] × 100, where A_1_ represents the absorbance of the sample solution, A_2_ represents the absorbance of the sample solution control, A_3_ represents the absorbance of blank, and A_0_ represents the absorbance of the blank control. The test of each concentration was performed three times parallelly. The fitting curve of inhibition rates vs. different concentrations of the test compound derived a half-maximal inhibitory concentration (IC_50_).

### 3.6. Molecular Docking Experiments

The software AutoDock Vina (v4.2) was employed for the molecular docking studies. The crystal structure of the mushroom tyrosinase with PDB No. 2Y9X was obtained from the RCSB Protein Data Bank. Removal of the ligand and water molecules from the crystal structure was conducted by the software Pymol (v2.4). The software AutoDock Vina (v4.2) detected the active site of the crystal structure and set the coordinates to (−7.40 −23.55 −32.51) and the grid box size at 70.36 Å × 70.36 Å × 70.36 Å. The software Discovery Studio Visualizer (v19.1) was used for the analysis.

## 4. Conclusions

In summary, a detailed chemical investigation of the ornamental and medicinal plant *L. japonicum* led to the isolation and identification of an array of components with various structural characteristics, including seven serratane-type triterpenes **1**–**7**, one steroid **8**, five benzene derivatives **9**–**13**, and two furan derivatives **14** and **15**. Among them, compounds **3**, **5** and **9**–**15** were reported from the plant *L. japonicum* for the first time. All the isolates except for the previously investigated **6**–**9** were assessed for tyrosinase inhibitory activity. As a result, compounds **4**, **12**, **13**, **14**, and **15** exhibited significant inhibition against tyrosinase (IC_50_ values ranging from 1.46 mM to 6.82 mM), which were close in value to the positive control Kojic acid (IC_50_ = 0.17 mM). This work not only enriched the chemical warehouse of the species *L. japonicum* but also supplemented the foundation for the future cosmetical and medicinal utilization of this species.

## Figures and Tables

**Table 1:** The identification of compounds **1**–**15** from *L. japonicum*.

Compounds	Names	References
**1**	16-oxo-3α-hydroxyserrat-14-en-21β-ol	[16]
**2**	21-*epi*-serratenediol	[24]
**3**	3β-hydroxy-14-serraten-21-one	[25]
**4**	serratenediol	[24,26]
**5**	methyl lycernuate A	[27,28]
**6**	α-onocerin	[29]
**7**	26-nor-8-oxo-α-onocerin	[30]
**8**	β-stiosterol	[23]
**9**	*trans*-ethyl ferulate	[31]
**10**	*cis*-ethyl ferulate	[31,32]
**11**	ethyl 4-hydroxy-3-methoxybenzoate	[33]
**12**	2,4-di-*tert*-butylphenol	[34]
**13**	ginkwanghol A	[35]
**14**	5-ethoxymethylfurfural	[36]
**15**	5-hydroxymethyl furfural	[37]

**Table 2:** The tyrosinase inhibitory results of compounds **4**, **12**, **13**, **14,** and **15** and the positive control Kojic acid.

Compounds	IC_50_ (mM)
**4**	2.16 ± 0.03
**12**	3.28 ± 0.03
**13**	1.46 ± 0.03
**14**	5.92 ± 0.06
**15**	6.82 ± 0.09
Kojic acid ^1^	0.17 ± 0.01

^1^ Positive control.

## Data Availability

Data is contained within the article.

## Supplementary Materials

The following supporting information can be downloaded at https://www.mdpi.com/article/10.3390/molecules30194024/s1, Tables S1–S15: The ^1^H and ^13^C NMR data of compound **1**–**15** and comparison with the data in the literature.
