# Supplementary material for: Natural Tyrosinase Inhibitors from Lycopodium japonicum
Source: Molecules. 2025 Oct 9;30(19):4024. doi: 10.3390/molecules30194024 (PMC12525909; doi:10.3390/molecules30194024)
Supplement: Supplementary file 1 [file molecules-30-04024-s001.zip › molecules-3789773-supplementary.pdf]

Supplementary Materials

1. Comparison of the ^1H and ^{13}C NMR data between isolated and reported data

Table S1. The ^1H and ^{13}C NMR data of compound **1** and comparison with the data in the literature.
Table S2. The ^1H and ^{13}C NMR data of compound **2** and comparison with the data in the literature.
Table S3. The ^1H and ^{13}C NMR data of compound **3** and comparison with the data in the literature.
Table S4. The ^1H and ^{13}C NMR data of compound **4** and comparison with the data in the literature.
Table S5. The ^1H and ^{13}C NMR data of compound **5** and comparison with the data in the literature.
Table S6. The ^1H and ^{13}C NMR data of compound **6** and comparison with the data in the literature.
Table S7. The ^1H and ^{13}C NMR data of compound **7** and comparison with the data in the literature.
Table S8. The ^1H and ^{13}C NMR data of compound **8** and comparison with the data in the literature.
Table S9. The ^1H and ^{13}C NMR data of compound **9** and comparison with the data in the literature.
Table S10. The ^1H and ^{13}C NMR data of compound **10** and comparison with the data in the literature.
Table S11. The ^1H and ^{13}C NMR data of compound **11** and comparison with the data in the literature.
Table S12. The ^1H and ^{13}C NMR data of compound **12** and comparison with the data in the literature.
Table S13. The ^1H and ^{13}C NMR data of compound **13** and comparison with the data in the literature.
Table S14. The ^1H and ^{13}C NMR data of compound **14** and comparison with the data in the literature.
Table S15. The ^1H and ^{13}C NMR data of compound **15** and comparison with the data in the literature.

2. References

Table S1. The ^{1}H and ^{13}C NMR data of compound **1** and comparison with the data in the literature.

**Position**	**1** [1]		16-oxo-3α-hydroxyserrat-14-en-21β-ol	
	δ_H (ppm, *J* in Hz)	δ_C (ppm)	δ_H (ppm, *J* in Hz) (Ref. [1] [1])	δ_C (ppm) (Ref. [1] [1])
1		33.3		32.9
2		26.6		26.2
3	3.41 (t, 2.9)	76.9	3.42 (br s)	75.8
4		38.4		38.6
5		49.4		48.9
6		18.8		18.4
7		45.0		44.6
8		38.2		38.2
9		62.6		62.1
10		37.8		38.0
11		24.7		24.4
12		25.6		25.2
13		59.0		58.5
14		163.7		164.2
15	5.71 (t, 2.5)	128.8	5.72 (br s)	128.1
16		201.4		202.0
17		58.8		58.3
18		44.4		44.2
19		31.6		31.2
20		25.2		24.7
21	3.35 (t, 2.9)	76.1	3.36 (br s)	75.2
22		36.9		37.6
23		28.5		28.1
24		22.3		21.9
25		15.8		15.3
26		20.1		19.6
27		56.0		55.6
28		15.0		14.5
29		21.6		21.3
30		28.0		27.8

[1] Measured in $CDCl_3$.

Table S2. The ^{1}H and ^{13}C NMR data of compound **2** and comparison with the data in the literature.

Position	**2** [1]		21-*epi*-serrate ediol	
	δ_H (ppm, *J* in Hz)	δ_C (ppm)	δ_H (ppm, *J* in Hz) (Ref. [2] [1])	δ_C (ppm) (Ref. [2] [1])
1		38.8		38.6
2		27.3		27.2
3	3.18 (dd, 11.7, 4.6)	79.0	3.18 (dd, 11.6, 4.7)	78.8
4		38.3		38.2
5		55.9		55.7
6		19.1		18.9
7		45.3		45.2
8		37.6		37.4
9		63.1		62.9
10		39.1		39.0
11		25.4		25.2
12		27.7		27.5
13		57.0		56.8
14		138.7		138.5
15	5.32 (br s)	122.2	5.33 (br s)	122.1
16		24.2		24.0
17		43.5		43.4
18		36.1		35.9
19		31.4		31.2
20		25.6		25.4
21	3.45 (t, 2.9)	76.4	3.45 (br s)	76.2
22		37.3		37.1
23	0.80 (s)	28.3	0.80 (s)	28.1
24	0.88 (s)	15.6	0.89 (s)	15.4
25	0.77 (s)	15.9	0.77 (s)	15.7
26	0.84 (s)	19.9	0.84 (s)	19.8
27		56.4		56.2
28	0.69 (s)	13.4	0.69 (s)	13.3
29	0.93 (s)	21.9	0.93 (s)	21.8
30	0.97 (s)	27.9	0.97 (s)	27.7

[1] Measured in $CDCl_3$.

Table S3. The ^1H and ^{13}C NMR data of compound **3** and comparison with the data in the literature.

**Position**	**3** [1]		3β-hydroxy-14-serraten-21-one	
	δ_H (ppm, J in Hz)	δ_C (ppm)	δ_H (ppm, J in Hz) (Ref. [3] [1])	δ_C (ppm) (Ref. [3] [1])
1		38.7		38.6
2		27.7		27.5
3	3.19 (dd, 11.7, 4.6)	79.0	3.19 (dd, 11.5, 4.5)	78.8
4		38.3		38.2
5		51.3		51.2
6		19.0		18.9
7		45.3		45.1
8		37.2		37.1
9		62.9		62.7
10		39.1		39.0
11		25.6		25.5
12		27.3		27.2
13		56.6		56.5
14		138.5		138.3
15	5.38 (br s)	122.1	5.38 (s)	122.0
16		24.7		24.5
17		55.9		55.7
18		36.3		36.2
19		34.9		34.8
20		38.5		38.4
21		217.2		217.0
22		47.8		47.7
23	0.97 (s)	28.3	0.97 (s)	28.1
24	0.77 (s)	15.6	0.77 (s)	15.4
25	0.80 (s)	15.9	0.80 (s)	15.7
26	0.83 (s)	19.9	0.83 (s)	19.8
27		56.0		55.9
28	0.92 (s)	13.1	0.92 (s)	12.9
29	1.04 (s)	24.6	1.04 (s)	24.5
30	1.08 (s)	21.7	1.09 (s)	21.6

[1] Measured in $CDCl_3$.

Table S4. The ^{1}H and ^{13}C NMR data of compound **4** and comparison with the data in the literature.

Position	**4** [1]		serratenediol	
	δ_H (ppm, *J* in Hz)	δ_C (ppm)	δ_H (ppm, *J* in Hz) (Ref. [2] [1])	δ_C (ppm) (Ref. [4] [1])
1		38.7		38.7
2		27.8		27.8
3	3.19 (dd, 11.7, 4.6)	79.0	3.19 (dd, 11.5, 4.1)	78.9
4		39.0		39.0
5		55.8		55.8
6		19.0		19.0
7		45.3		45.3
8		37.2		37.2
9		63.0		62.9
10		38.3		38.3
11		25.4		25.4
12		27.4		27.4
13		57.3		57.3
14		138.3		138.3
15	5.33 (br s)	122.3	5.32 (br s)	122.3
16		24.2		24.2
17		49.6		49.6
18		36.3		36.3
19		37.3		37.3
20		27.7		27.7
21	3.23 (dd, 11.6, 4.2)	79.3	3.23 (dd, 11.4, 4.1)	79.3
22		39.1		39.1
23	0.80 (s)	15.6	0.80 (s)	15.6
24	0.83 (s)	28.3	0.83 (s)	28.2
25	0.77 (s)	15.9	0.77 (s)	15.9
26	0.83 (s)	19.9	0.83 (s)	19.9
27		56.2		56.2
28	0.67 (s)	13.6	0.67 (s)	13.5
29	0.96 (s)	14.8	0.96 (s)	14.7
30	0.97 (s)	27.7	0.97 (s)	27.7

[1] Measured in $CDCl_3$.

Table S5. The ^{1}H and ^{13}C NMR data of compound **5** and comparison with the data in the literature.

Position	**5** [1]		lycernuic acid A		lycernuic ketone A
	δ_H (ppm, *J* in Hz)	δ_C (ppm)	δ_H (ppm, *J* in Hz) (Ref. [5] [1])	δ_C (ppm) (Ref. [5] [1])	δ_C (ppm) (Ref. [5] [1])
1		39.8		40.0	39.6
2		29.3		29.8	29.1
3	3.36 (dd, 12.2, 4.6)	78.4	3.38 (dd, 12.0, 4.2)	78.5	78.2
4		50.2		49.7	50.0
5		57.0		57.3	56.9
6		21.4		21.6	21.1
7		45.7		45.9	45.4
8		37.6		37.6	38.1
9		62.9		62.9	62.4
10		39.0		39.3	38.9
11		27.0		27.0	25.7
12		28.1		28.1	30.3
13		57.7		57.6	59.4
14		139.8		139.3	164.4
15	5.51 (br s)	123.3	5.50 (br s)	123.2	129.3
16		25.0		24.9	202.3
17		44.2		44.1	60.6
18		36.8		36.7	45.1
19		32.2		32.2	32.3
20		26.0		26.0	26.2
21	3.70 (br s)	75.6	3.68 (br s)	75.6	70.3
22		38.4		38.3	44.1
23	1.58 (s)	24.6	1.72 (s)	25.1	24.5
24		178.4		181.1	177.8
25	0.80 (s)	14.1	1.05 (s)	14.6	14.1
26	0.88 (s)	20.1	0.89 (s)	20.1	20.0
27		57.2		57.1	56.1
28	0.85 (s)	14.2	0.85 (s)	14.2	16.2
29	0.97 (s)	22.5	0.96 (s)	22.5	64.5
30	1.18 (s)	29.1	1.16 (s)	29.0	23.3
OCH_3	3.64 (s)	51.4			51.4

[1] Measured in C_5D_5N.

Table S6. The ^{1}H and ^{13}C NMR data of compound **6** and comparison with the data in the literature.

Position	**6** [1]		α-onocer n	
	δ_H (ppm, *J* in Hz)	δ_C (ppm)	δ_H (ppm, *J* in Hz) (Ref. [6] [1])	δ_C (ppm) (Ref. [6] [1])
1		37.2		37.2
2		28.1		28.1
3	3.25 (dd, 11.7, 4.2)	79.1	3.26 (dd, 11.7, 4.9)	79.1
4		39.4		39.4
5		54.8		54.8
6		24.2		24.2
7		38.4		38.4
8		148.6		148.6
9		57.7		57.7
10		39.3		39.3
11		22.8		22.8
12		22.8		22.8
13		57.7		57.7
14		148.6		148.6
15		38.4		38.4
16		24.2		24.2
17		54.8		54.8
18		39.3		39.3
19		37.2		37.2
20		28.1		28.1
21	3.25 (dd, 11.7, 4.2)	79.1	3.26 (dd, 11.7, 4.9)	79.1
22		39.4		39.4
23	0.99 (s)	28.5	0.99 (s)	28.5
24	0.76 (s)	15.5	0.76 (s)	15.5
25	0.64 (s)	14.7	0.64 (s)	14.7
26	4.83 (s), 4.56 (s)	106.9	4.84 (s), 4.56 (s)	106.8
27	4.83 (s), 4.56 (s)	106.9	4.84 (s), 4.56 (s)	106.8
28	0.64 (s)	14.7	0.64 (s)	14.7
29	0.76 (s)	28.5	0.76 (s)	28.5
30	0.99 (s)	15.5	0.99 (s)	15.5

[1] Measured in $CDCl_3$.

Table S7. The ^{1}H and ^{13}C NMR data of compound **7** and comparison with the data in the literature.

Position	**7** [1]		26-nor-8-oxo-α-onocerin	
	δ_H (ppm, *J* in Hz)	δ_C (ppm)	δ_H (ppm, *J* in Hz) (Ref. [7] [1])	δ_C (ppm) (Ref. [7] [1])
1		37.1		37.1
2		28.5		28.4
3	3.34 (dd, 11.7, 4.0)	78.8	3.33 (dd, 11.7, 4.1)	78.7
4		39.3		39.3
5		54.8		54.8
6		27.7		27.7
7		42.5		42.5
8		212.1		212.2
9		64.8		64.8
10		42.4		42.4
11		21.2		21.2
12		23.7		23.7
13		57.4		57.3
14		147.5		147.5
15		37.3		37.3
16		23.7		23.7
17		53.7		53.7
18		38.4		38.3
19		37.1		37.1
20		28.1		28.0
21	3.23 (dd, 11.8, 4.2)	79.1	3.23 (dd, 11.7, 4.2)	79.1
22		39.4		39.4
23	0.69 (s)	27.7	0.69 (s)	27.7
24	0.81 (s)	15.5	0.81 (s)	15.5
25	1.09 (s)	14.6	1.08 (s)	14.6
26				
27	4.91 (s), 4.89 (s)	108.0	4.90 (s) × 2	108.0
28	0.99 (s)	14.9	0.98 (s)	14.9
29	0.63 (s)	28.5	0.63 (s)	28.5
30	0.76 (s)	15.5	0.75 (s)	15.5

[1] Measured in $CDCl_3$.

Table S8. The ^{1}H and ^{13}C NMR data of compound **8** and comparison with the data in the literature.

**Position**	**8** [1]		*β*-stiosteıol	
	δ_H (ppm, *J* in Hz)	δ_C (ppm)	δ_H (ppm, *J* in Hz) (Ref. [8] [1])	δ_C (ppm) (Ref. [8] [1])
1		37.4		37.4
2		32.1		32.1
3	3.52 (m)	72.0	3.52 (td, 11.1, 5.6)	72.0
4		42.4		42.4
5		140.9		140.9
6	5.35 (m)	121.9	5.35 (m)	121.9
7		32.1		32.1
8		31.8		31.8
9		50.3		50.3
10		36.7		36.7
11		21.2		21.2
12		39.9		39.9
13		42.5		42.5
14		56.9		56.9
15		24.5		24.5
16		28.4		28.4
17		56.2		56.2
18	0.68 (s)	12.0	0.68 (s)	12.0
19	1.01 (s)	19.2	1.01 (s)	19.2
20		36.3		36.3
21	0.92 (d, 6.6)	18.9	0.92 (d, 6.5)	18.9
22		34.1		34.1
23		26.2		26.2
24		46.0		46.0
25		29.3		29.3
26	0.83 (d, 6.7)	19.5	0.83 (d, 6.8)	19.6
27	0.81 (d, 6.8)	20.0	0.81 (d, 6.8)	20.0
28		23.2		23.2
29	0.84 (t, 7.7)	12.1	0.84 (t, 7.5)	12.1

[1] Measured in $CDCl_3$.

Table S9. The ^{1}H and ^{13}C NMR data of compound **9** and comparison with the data in the literature.

Position	**9** [1]		*trans*-ethyl fe ulate	
	δ_H (ppm, *J* in Hz)	δ_C (ppm)	δ_H (ppm, *J* in Hz) (Ref. [9] [1])	δ_C (ppm) (Ref. [9] [1])
1		127.2		126.9
2	7.03 (d, 1.9)	109.4	7.02 (d, 1.8)	109.3
3		148.0		147.9
4		146.9		146.8
5	6.92 (d, 8.2)	114.8	6.91 (d, 8.4)	114.7
6	7.07 (dd, 8.2, 2.0)	123.2	7.06 (dd, 8.4, 1.8)	123.0
1'	7.61 (d, 15.9)	144.8	7.61 (d, 15.9)	144.7
2'	6.29 (d, 15.9)	115.8	6.29 (d, 15.9)	115.5
3'		167.4		167.3
4'	4.26 (q, 7.1)	60.5	4.26 (q, 6.9)	60.3
5'	1.33 (t, 7.1)	14.5	1.33 (t, 6.9)	14.3
OCH_3	3.93 (s)	56.1	3.91 (s)	55.8

[1] Measured in $CDCl_3$.

Table S10. The ^1H and ^{13}C NMR data of compound **10** and comparison with the data in the literature.

Position	**10** [1]		amyl (*Z*)-ferulic acid ester	
	δ_H (ppm, *J* in Hz)	δ_C (ppm)	δ_H (ppm, *J* in Hz) (Ref. [10] [1])	δ_C (ppm) (Ref. [10] [1])
1		127.4		127.4
2	7.77 (d, 2.0)	112.9	7.76 (d, 1.9)	112.9
3		146.0		146.0
4		147.2		147.1
5	6.88 (d, 8.2)	113.9	6.90 (d, 8.1)	113.9
6	7.11 (dd, 8.3, 2.0)	125.8	7.10 (dd, 8.1, 1.9)	125.7
1'	6.79 (d, 12.9)	143.8	6.79 (d, 12.8)	143.7
2'	5.81 (d, 12.9)	117.0	5.82 (d, 12.8)	117.1
3'		166.7		166.8
4'	4.19 (q, 7.1)	60.3	4.12 (t, 6.8)	64.6
5'	1.29 (t, 7.2)	14.4	1.65 (m)	28.5
6'			1.33 (m)	28.3
7'			1.33 (m)	22.5
8'			0.90 (t, 6.8)	14.4
OCH_3	3.93 (s)	56.1	3.93 (s)	56.1
OH	5.82 (br s)		5.82 (br s)	

[1] Measured in $CDCl_3$.

Table S11. The ^{1}H and ^{13}C NMR data of compound **11** and comparison with the data in the literature.

**Position**	**11** [1]		ethyl 4-hydroxy-3-methoxybenzoate	
	δ_H (ppm, *J* in Hz)	δ_C (ppm)	δ_H (ppm, *J* in Hz) (Ref. [11] [1])	δ_C (ppm) (Ref. [11] [1])
1		124.2		124.3
2	7.55 (d, 1.9)	111.8	7.55 (d, 2.0)	111.9
3		146.3		146.3
4		150.1		150.1
5	6.93 (d, 8.3)	114.1	6.94 (d, 8.5)	114.2
6	7.64 (dd, 8.3, 1.9)	122.8	7.64 (dd, 8.5, 2.0)	122.9
1'		166.6		166.6
2'	4.35 (q, 7.1)	60.9	4.35 (q, 7.2)	60.9
3'	1.38 (t, 7.1)	14.5		14.5
OCH_3	3.95 (s)	56.2	3.94 (s)	56.3

[1] Measured in $CDCl_3$.

Table S12. The ^1H and ^{13}C NMR data of compound **12** and comparison with the data in the literature.

Position	**12** [1]		2,4-di-*tert*-butylphenol	
	δ_H (ppm, J in Hz)	δ_C (ppm)	δ_H (ppm, J in Hz) (Ref. [12] [1])	δ_C (ppm) (Ref. [12] [1])
1		151.9		151.7
2		143.1		142.9
3	7.31 (d, 2.4)	116.1	7.31 (d, 2.4)	115.9
4		135.3		135.1
5	7.08 (dd, 8.2, 2.4)	123.7	7.08 (dd, 8.2, 2.4)	123.5
6	6.60 (d, 8.2)	124.2	6.60 (d, 8.2)	124.5
7		34.4		34.3
8	1.30 (s)	31.8	1.30 (s)	31.6
9	1.30 (s)	31.8	1.30 (s)	31.6
10	1.30 (s)	31.8	1.30 (s)	31.6
11		34.9		34.7
12	1.43 (s)	29.8	1.43 (s)	29.6
13	1.43 (s)	29.8	1.43 (s)	29.6
14	1.43 (s)	29.8	1.43 (s)	29.6

[1] Measured in $CDCl_3$.

Table S13. The ^{1}H and ^{13}C NMR data of compound **13** and comparison with the data in the literature.

Position	**13** [1]		ginkwanghol A	
	δ_H (ppm, J in Hz)	δ_C (ppm)	δ_H (ppm, J in Hz) (Ref. [13] [1])	δ_C (ppm) (Ref. [13] [1])
1		161.3		159.9
2	6.82 (d, 8.6)	116.8	6.83 (d, 8.5)	115.4
3	7.48 (d, 8.6)	131.1	7.48 (d, 8.5)	129.7
4		127.2		125.8
5	7.48 (d, 8.6)	131.1	7.48 (d, 8.5)	129.7
6	6.82 (d, 8.6)	116.8	6.83 (d, 8.5)	115.4
1'	7.61 (d, 15.9)	146.3	7.62 (d, 16.0)	144.9
2'	6.33 (d, 15.9)	115.6	6.34 (d, 16.0)	114.2
3'		169.3		167.8
4'	5.01 (m)	75.4	5.00 (m)	73.9
5'	1.63 (m)	35.4	1.63 (m)	33.9
6'		26.5		24.9
7'		30.7		29.1
8'		26.9		25.2
9'	1.53 (m)	33.7	1.53 (m)	32.1
10'	3.55 (t, 6.6)	63.0	3.54 (t, 6.5)	61.5
11'	1.63	35.4	1.63	33.9
12'		26.4		24.9
13'		30.6		29.1
14'		30.6		29.1
15'		30.6		29.1
16'		30.5		29.1
17'		26.8		25.2
18'	1.53 (m)	33.5	1.53 (m)	32.1
19'	3.54 (t, 6.6)	62.9	3.54 (t, 6.5)	61.5

[1] Measured in CD_3OD.

Table S14. The ^{1}H and ^{13}C NMR data of compound **14** and comparison with the data in the literature.

**Position**	**14** [1]		5-ethoxymethylfurfural	
	δH (ppm, *J* in Hz)	δC (ppm)	δH (ppm, *J* in Hz) (Ref. [14] [1])	δC (ppm) (Ref. [14] [1])
1	9.62 (s)	177.9	9.59 (s)	177.6
2		158.9		158.7
3	6.52 (d, 3.6)	122.1	6.50 (d, 3.5)	121.8
4	7.21 (d, 3.5)	111.1	7.19 (d, 3.5)	110.9
5		152.7		152.5
6	4.53 (s)	66.8	4.50 (s)	66.5
7	3.59 (q, 7.0)	64.9	3.58 (q, 7.1)	64.7
8	1.24 (t, 7.0)	15.2	1.21 (t, 7.1)	15.0

[1] Measured in $CDCl_3$.

Table S15. The ^{1}H and ^{13}C NMR data of compound **15** and comparison with the data in the literature.

Position	**15** [1]		5-hydroxymethyl furfural	
	δ_H (ppm, *J* in Hz)	δ_C (ppm)	δ_H (ppm, *J* in Hz) (Ref. [15] [1])	δ_C (ppm) (Ref. [15] [1])
1	9.60 (s)	177.8	9.57 (s)	177
2		160.6		160
3	6.52 (d, 3.5)	122.3	6.53 (d, 4.0)	122
4	7.21 (d, 3.5)	110.1	7.23 (d, 4.0)	109
5		152.6		152
6	4.72 (s)	57.8	4.72 (s)	57

[1] Measured in $CDCl_3$.

References

1. Li, M.-j.; Liu, J.; Zhang, Y.-b.; Chen, N.-h.; Wang, G.-c.; Li, Y.-l. Chemical constituents from whole herb of *Lycopodium japonicum*. *Chin. Tradit. Herb. Drugs* **2015**, *46*, 33-37. https://doi.org/10.7501/j.issn.0253-2670.2015.01.008.
2. Yang, G.-X.; Zang, Y.; Hu, C.-L.; Xiong, J.; Hu, J.-F. Serratene triterpenoids in *Lycopodium japonicum* from Hubei province. *Chin. Tradit. Herb. Drugs* **2014**, *45*, 3524-3527. https://doi.org/10.7501/j.issn.0253-2670.2014.24.004.
3. Sa, N.H.; Tam, N.T.; Quan, T.D.; Tinh, B.X.; Thien, D.D.; Sung, T.V.; Adorisio, S.; Delfino, D.V. Chemical constituents and their biological activity of *Pinus dalatensis*. Part 1. Terpenoids from the leaves. *Vietnam J. Chem.* **2017**, *55*, 509-513. https://doi.org/10.15625/2525-2321.2017-004500.
4. Zheng, K.-k.; Zhao, Y.-y.; Yuan, P.-l.; Wu, Y.-c.; Zhang, L.-q.; Guo, F.-j.; Li, Y.-m. Chemical constituents of non-alkaloids from *Huperzia serrata*. *Chin. Tradit. Herb. Drugs* **2016**, *47*, 15-20. https://doi.org/10.7501/j.issn.0253-2670.2016.01.003.
5. Zhang, Z.; ElSohly, H.N.; Jacob, M.R.; Pasco, D.S.; Walker, L.A.; Clark, A.M. Natural products inhibiting *Candida albicans* secreted aspartic proteases from *Lycopodium cernuum*. *J. Nat. Prod.* **2002**, *65*, 979-985. https://doi.org/10.1021/np0200616.
6. Zhu, M.; Wang, W.-y.; Chen, W.-j.; Wei, J.-h.; Liang, C.-y.; Feng, X.; Lu, C.-s. Chemical constituents and antioxidant activity *in vitro* of Zhuang medicine *Dalbergia rimosa*. *J. Chin. Med. Mater.* **2024**, *47*, 1147-1152. https://doi.org/10.13863/j.issn1001-4454.2024.05.013.
7. Teng, X.-f.; Liu, Y.-l.; Yang, F.; Wang, H.-j.; He, L. Chemical constituents from *Lycopodium fargesii* and their anti-osteoporotic activity. *Nat. Prod. Res. Dev.* **2022**, *34*, 226-231. https://doi.org/10.16333/j.1001-6880.2022.2.007.
8. Bu, Q.; Ge, Z.-Y.; Liang, L.-F. Chemical and biological investigation on the potential ornamental plant *Ophiorrhiza chinensis*. *Agronomy* **2024**, *14*, 1872. https://doi.org/10.3390/agronomy14081872.
9. Pal, P.P.; Begum, S.A.; Basha, A.S.; Araya, H.; Fujimoto, Y. A new lignan (polonilignan) and inhibitors of nitric oxide production from *Penicillium polonicum*, an endophytic fungi of *Piper nigrum*. *Chem. Biodivers.* **2023**, *20*, e202200840. https://doi.org/10.1002/cbdv.202200840.
10. Cao, X.-X.; Sun, J.-Y.; Liu, C.; Zhang, J.-S.; Zhang, H. Antiradical aromatic constituents from *Pleurotus eryngii*. *Rec. Nat. Prod.* **2021**, *15*, 169-174. https://doi.org/10.25135/rnp.205.20.08.1789.
11. Li, Y.; Zhou, B.-p.; Zhang, W.-j.; Yang, G.-l.; Zhang, C.-l.; Cao, Z.-y. Chemical constituents from aerial parts of *Ribes mandshuricum*. *Chin. Tradit. Herb. Drugs* **2018**, *49*, 772-779. https://doi.org/10.7501/j.issn.0253-2670.2018.04.003.
12. Cho, J.-Y.; Kim, M.-S.; Lee, Y.G.; Jeong, H.Y.; Lee, H.J.; Ham, K.-S.; Moon, J.-H. A phenyl lipid alkaloid and flavone *C*-diglucosides from *Spergularia marina*. *Food Sci. Biotechnol.* **2016**, *25*, 63-69. https://doi.org/10.1007/s10068-016-0009-7.
13. Lee, K.H.; Kim, J.K.; Yu, J.S.; Jeong, S.Y.; Choi, J.H.; Kim, J.-C.; Ko, Y.-J.; Kim, S.-H.; Kim, K.H. Ginkwanghols A and B, osteogenic coumaric acid-aliphatic alcohol hybrids from the leaves of *Ginkgo biloba*. *Arch. Pharm. Res.* **2021**, *44*, 514-524. https://doi.org/10.1007/s12272-021-01329-3.
14. Bredihhin, A.; Mäeorg, U.; Vares, L. Evaluation of carbohydrates and lignocellulosic biomass from different wood species as raw material for the synthesis of 5-bromomethyfurfural. *Carbohydr. Res.* **2013**, *375*, 63-67. https://doi.org/10.1016/j.carres.2013.04.002.
15. Miyazawa, M.; Anzai, J.; Fujioka, J.; Isikawa, Y. Insecticidal compounds against *Drosophila melanogaster* from *Cornus officinalis* Sieb. Et Zucc. *Nat. Prod. Res.* **2003**, *17*, 337-339. https://doi.org/10.1080/1057563031000072587.
